# Decades in the Making: A Rare Case of Mastoid Squamous Cell Carcinoma Following Chronic Otitis Media and Mastoidectomy

**DOI:** 10.7759/cureus.95005

**Published:** 2025-10-20

**Authors:** Mikayla Musumeci, Zachary B Bonitz, Brandon T Abrenica, John F Ferguson, David E Rubin

**Affiliations:** 1 Internal Medicine, Touro College of Osteopathic Medicine, Middletown, USA; 2 Pulmonology and Critical Care, Garnet Health Medical Center, Middletown, USA; 3 Pathology, Montefiore St. Luke's Cornwall, Cornwall, USA

**Keywords:** chronic otitis media (com), mastoidectomy, mastoiditis, nonkeratinizing squamous cell carcinoma (nkscc), temporal bone squamous cell carcinoma (tbscc)

## Abstract

Squamous cell carcinoma (SCC) of the mastoid, a subtype of temporal bone SCC (TBSCC), is a rare and aggressive malignancy often associated with chronic otologic conditions such as chronic otitis media (COM) and mastoiditis. We present the case of a 67-year-old male with a lifelong history of COM and a childhood mastoidectomy who developed SCC of the right mastoid decades later. The patient developed worsening otologic symptoms, including persistent bloody ear discharge that ultimately led to diagnosis via mastoid biopsy. Surgical resection revealed poorly differentiated SCC involving the mastoid, middle ear, and adjacent structures, with positive margins due to tumor adherence to the dura. The patient was treated with adjuvant radiation and cisplatin-based chemotherapy, resulting in no evidence of recurrence or metastasis at follow-up.

This case shows that chronic ear disease can, in rare instances, progress to malignancy and reminds clinicians to stay alert when otologic symptoms worsen or persist. It also illustrates the importance of early biopsy and imaging in differentiating benign chronic inflammation from malignant transformation. Despite incomplete resection, the patient’s favorable response to multiple forms of therapy suggests that aggressive combined treatment can still give meaningful disease control. Increased awareness, early diagnosis, and comprehensive management strategies are critical to improving outcomes in patients at risk for TBSCC.

## Introduction

Temporal bone malignancies make up only 0.2% of all head and neck cancers, with SCC representing about 39% of those cases [1-3]. TBSCC is a low-incidence but high-risk malignancy, often arising in the setting of chronic ear disease [1,3,4]. This report presents a 67-year-old male with a lifelong history of COM and mastoiditis culminating in the diagnosis of SCC of the right mastoid decades after initial mastoidectomy. The patient's prolonged course of symptoms, from childhood through late adulthood, demonstrates the diagnostic challenges and potential for malignant transformation in patients who have chronically inflamed middle ear disorders [5,6]. This case serves as a reminder that persistent otologic symptoms require careful follow-up, and it provides insight into management strategies for SCC that involve important neurologic and otologic structures [6].

## Case presentation

**Figure 1:**
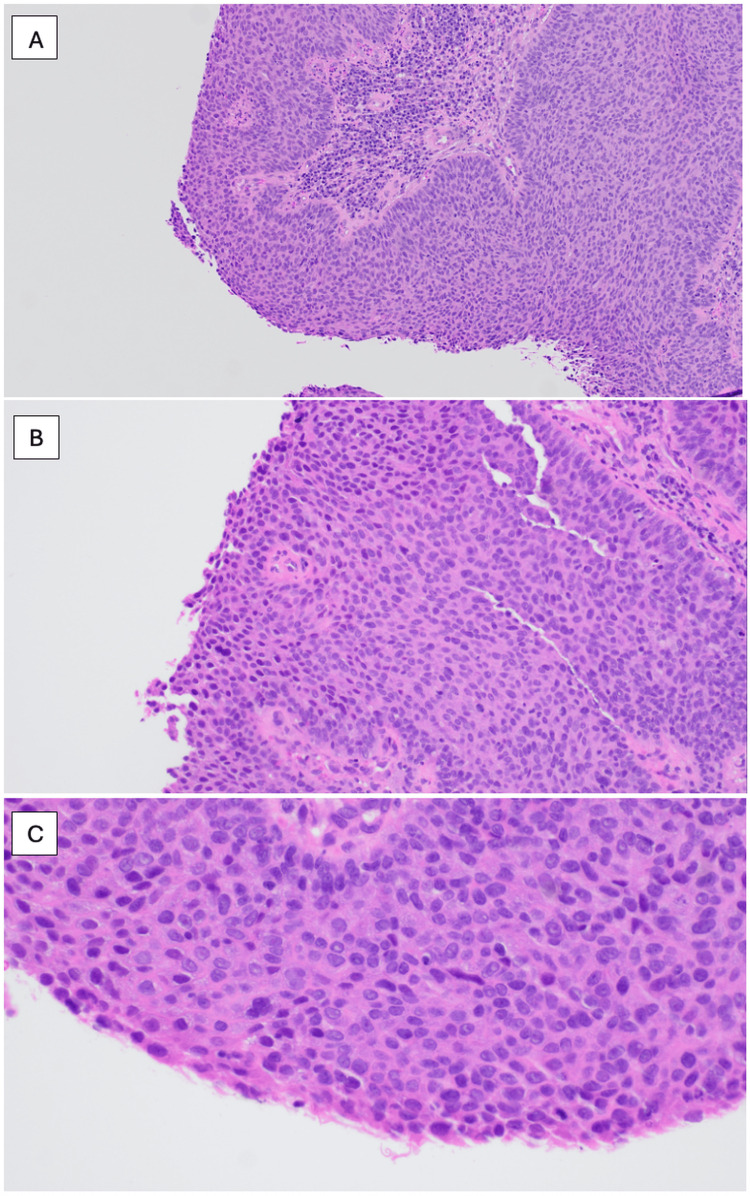
H&E-stained section of right mastoid biopsy (2023) confirming poorly differentiated squamous cell carcinoma. (A) x10, (B) x20, (C) x40.

**Figure 2:**
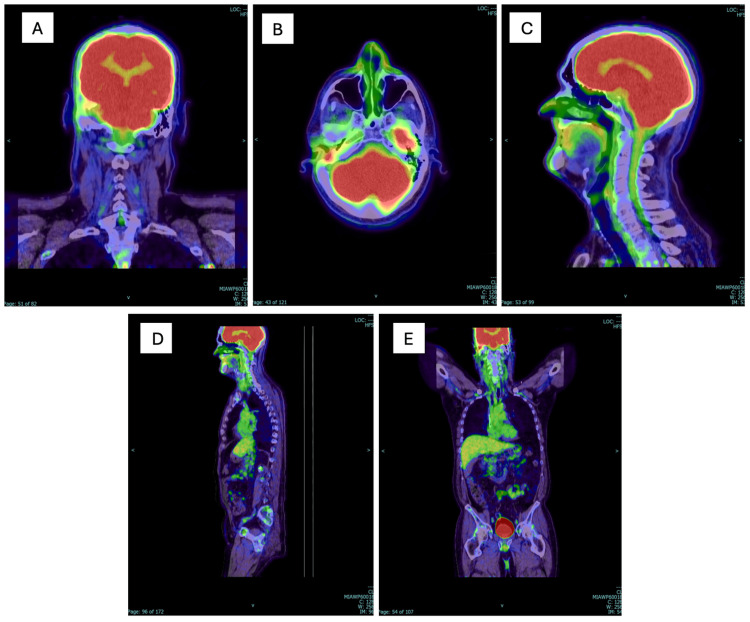
Follow-up PET/CT from skull base to mid-thigh performed in 2024. (A) Fused coronal images of the head and neck. (B) Fused axial images of the head and neck. (C) Fused sagittal image of the head and neck. (D) & (E) Sagittal and coronal fused images. PET/CT: Positron emission tomography/computed tomography

This case involves a 67-year-old male with a history of COM and mastoiditis, leading to a right mastoidectomy in 1965 and, ultimately, a diagnosis of SCC of the right temporal bone/mastoid. The patient’s symptoms included headaches, postauricular swelling, and ear drainage that began in childhood. This led to a right mastoidectomy at the age of eight, resulting in significant hearing loss. Following mastoidectomy, he continued to experience COM, hearing loss, tinnitus, eustachian tube dysfunction, and purulent ear drainage. Of note, the patient was a heavy smoker and worked around cars, in factories, and did maintenance throughout his career. Smoking tobacco is a known risk factor for SCC of the head and neck, and possible industrial occupational exposures during the patient’s career may be linked with SCC. Persistent ear drainage throughout his life became more severe in 2022, characterized by constant bloody discharge. After multiple therapeutic mastoid debridements by neurotology involving the removal of debris, granulation tissue, and infection, followed by irrigation, persistent findings warranted further evaluation. In 2023, a CT of the temporal bone showed complete opacification of the canal wall cavity and dehiscence of bone in the region of the tegmen mastoideum on the right side. A subsequent mastoid biopsy confirmed the presence of SCC of the right mastoid. Surgical intervention involved subtotal resection of the right temporal bone, middle ear, and mastoid mass, with microscopic residual disease identified along the dura. Pathology revealed poorly differentiated SCC involving the right mastoid, middle ear, middle fossa, incus, and epitympanum (Figure 1). Although the entire mastoid cavity was surgically addressed, the tumor adhered to the mastoid dura, resulting in incomplete resection with positive surgical margins. Subsequent treatment included 35 sessions of radiation therapy (200 cGy per dose, totaling 7,000 cGy) and weekly chemotherapy with cisplatin (40 mg/m²). A follow-up CT/PET scan in 2024 showed no evidence of regional or distant metastasis (Figure 2). The patient continues to be followed closely by otolaryngology, oncology, and his primary care physician.

## Discussion

Primary TBSCC accounts for less than 0.25% of all head and neck cancers, most commonly presenting as the keratinizing subtype, while the non-keratinizing subtype is extremely rare [6]. Its development is often associated with chronic otologic conditions, such as long-standing COM and mastoiditis, as seen in this patient [1,3,4,7]. Although the pathophysiology of TBSCC is not fully understood, chronic inflammation and persistent infection have been common factors. 

A 2024 case report described a 63-year-old woman with a history of chronic suppurative otitis media who developed SCC of the middle ear that was successfully treated with partial removal of the petrous bone of the temporal bone and adjuvant chemoradiation [6-8]. Similarly, a case of an 80-year-old woman who developed TBSCC decades after an initial ear surgery and years of intermittent otorrhea secondary to chronic inflammation and cholesteatoma ultimately required radical radiation [1]. Another case series discussed three patients who initially presented with external auditory canal masses that were initially diagnosed as benign infections; one patient notably had undergone prior mastoid surgery and was later found to have moderately differentiated SCC involving the mastoid and middle ear [5]. Collectively, these cases reinforce that chronic otologic inflammation remains a significant risk factor for malignant transformation and demonstrate the importance of early biopsy and aggressive multimodal management.

In this case, the patient’s biopsy demonstrated non-keratinizing squamous cell carcinoma (NKSCC), which carries a poorer prognosis than the keratinizing subtype due to its more aggressive growth pattern and tendency for later detection. Histological features of NKSCC often demonstrate nests of tumor cells with pushing borders and comedo-type necrosis. In addition, there is an absence of stromal desmoplasia around the tumor nests [9]. High-power images of NKSCC show oval to spindled, hyperchromatic tumor cells that lack prominent nucleoli and have indistinct cell borders [10]. Several immunohistochemical stains are commonly used to identify and clarify NKSCC. Strong and diffuse positivity for p40, p63, and/or CK5/6 can confirm the squamous phenotype of the tumor [11]. Case reports have described the malignant transformation of benign HPV papilloma, so additional stain p16 can be used to further clarify the tumor based on HPV association [11]. 

Management of TBSCC typically involves aggressive surgical resection followed by adjuvant radiation therapy, with or without chemotherapy [6-8]. Complete surgical excision is the foundation of treatment; however, in cases where tumor adherence to critical structures (such as the mastoid dura) prevents complete resection, prognosis is significantly poorer [1,6]. A literature review of 16 case series, including 708 patients, found that 67% presented with advanced TBSCC, with most managed surgically (often lateral temporal bone resection) and adjunctive radiation used in all studies [12]. More than half of the studies reported 100% 2- to 5-year survival for T1-T2 disease without nodal involvement; however, advanced stage and poor differentiation were associated with poor prognosis, and postoperative radiotherapy improved survival in only one study [12]. An additional systematic review of 51 retrospective studies, including 501 patients with temporal bone SCC, found that those undergoing subtotal or total temporal bone resection had significantly higher rates of stage IV disease, positive margins, and recurrence compared with lateral resections [13]. Meta-analysis showed a 97% increase in mortality with subtotal/total resections, with recurrent disease and facial nerve involvement independently associated with decreased overall survival [13]. The positive margins in this patient's surgical pathology exemplify the challenge of achieving perfect resections in complex areas. However, the patient's successful response to adjuvant chemoradiation, with no evidence of recurrence or metastasis at follow-up, suggests that multimodal therapy can offer disease control even in cases with positive margins.

This case adds to existing concerns in the literature about the under-recognition of malignant transformation into chronic ear disease, and it indicates the need for earlier biopsy in clinically suspicious cases. It also supports the idea that multimodal treatment may improve outcomes even when complete resection is not possible. Previous research has investigated possible prognostic factors of patients with TBSCC; future clinical practice should prioritize early diagnosis and practicing multiple management strategies for patients with chronic otologic disease at risk for malignancy [14,15].

## Conclusions

This case highlights the rare but serious potential for SCC of the mastoid or temporal bone to develop decades after COM, mastoiditis, and mastoidectomy. Clinicians should maintain a high level of suspicion for malignancy in patients with persistent or changing otologic symptoms, especially when symptoms worsen after a history of chronic ear disease. Early biopsy and advanced imaging are important in distinguishing malignant from benign chronic infections. A combined treatment approach, including surgical resection and adjuvant chemoradiation, continues to be the standard of care, even when complete surgical excision is not successful. Future research should center on identifying early clinical and radiologic predictors of malignant transformation in chronic otologic conditions. Additionally, developing surveillance guidelines for high-risk patients to enable earlier diagnosis and improve outcomes would be beneficial to future patients. 
